# High-Purity Isolation of Polyphosphate-Rich Stabilisomes Defines Their Conserved Chemical Architecture in Thermophilic Cyanobacteria

**DOI:** 10.3390/plants15030499

**Published:** 2026-02-05

**Authors:** Chenyu Wang, Chuyuan Zhou, Xiaohua Song, Jingyun Yin, Mengmeng Wang, Liuyan Yang

**Affiliations:** State Key Laboratory of Water Pollution Control and Green Resource Recycling, Key Laboratory of Aquatic Ecosystem Health in the Middle and Lower Reaches of Yangtze River, Ministry of Ecology and Environment, School of Environment, Nanjing University, Nanjing 210023, China; 602022250043@smail.nju.edu.cn (C.W.); 502022250040@smail.nju.edu.cn (C.Z.); 502023250026@smail.nju.edu.cn (X.S.); yinjingyun@smail.nju.edu.cn (J.Y.)

**Keywords:** thermophilic cyanobacteria, polyphosphate, stabilisomes, purification methods, cyanobacterial resilience mechanisms

## Abstract

Thermophilic cyanobacteria are key models for thermotolerance and a promising source of thermophilic bioresources. Yet the subcellular basis of their stress resilience remains poorly resolved. Here, we focus on intracellular polyphosphate (polyP)-rich granules, termed “stabilisomes,” which have been implicated in stress adaptation. The lack of a high-purity, structure-preserving isolation method has been a major technical bottleneck hindering the elucidation of this resilience mechanism. This study describes a robust, structure-preserving purification strategy, boosting the granule-to-protein yield by over 10,000-fold compared with conventional methods. The specificity and structural integrity of this method are supported by the specific enrichment of complex proteomic (937 proteins) and metabolomic (1076 metabolites) signatures. Building on this, subsequent quantitative analysis across cyanobacteria at 7 hot spring sampling sites revealed a conserved core chemical composition dominated by polyphosphate (~21–36%), proteins (~10–20%), amino acids (~7–18%), and lipid components (~12–21%). The variability in abundance across species suggests a dynamic adjustment of these stabilizing components consistent with specific micro-environmental conditions. This work provides a robust bioseparation platform for prokaryotic organelles, offering a critical tool for investigating cyanobacterial resilience and developing novel biomaterials.

## 1. Introduction

The advancement of biotechnology increasingly relies on the development of efficient and scalable downstream processing technologies to recover high-value products from complex biological sources [1,2,3,4]. A persistent challenge in the field of bioseparations is the isolation of stress-responsive subcellular structures, such as organelles, while preserving their structural and functional integrity [5]. Geothermal environments, characterized by extreme heat, represent natural laboratories for studying the fundamental limits of life and the molecular strategies for survival under extreme heat [6,7]. In the context of these ecosystems, thermophilic cyanobacteria, which are found in geothermal environments, serve as an exceptional model for investigating microbial resilience under conditions of extreme environments [1,8,9]. Their inherent thermostability and robust cellular structures render them not only ecologically significant but also attractive chassis for biotechnological applications [1,10,11]. However, realizing their full potential is hindered by a two-fold bottleneck: a limited understanding of their unique intracellular adaptive mechanisms and the lack of specialized separation technologies capable of isolating these components from such robust organisms.

A key adaptation strategy observed in various cyanobacteria is the intracellular accumulation of inorganic polyphosphate (polyP), a multifunctional molecule implicated in phosphate storage, energy metabolism, and stress adaptation [12,13,14]. Studies have shown that various environmental stressors, such as high temperature and ultraviolet radiation, can trigger polyP synthesis in cyanobacteria, enhancing their resistance [15,16]. These polyP molecules are sequestered in electron-dense intracellular granules. Historically, these organelles are known by terms such as “volutin granules”, “polyphosphate granules”, or “acidocalcisomes”, which highlight their most prominent compositional features [17,18]. Notably, while these inclusions share significant physicochemical similarities with eukaryotic acidocalcisomes, we designate them as “stabilisomes” in this study to explicitly emphasize their distinct prokaryotic context and their specialized physiological function in conferring thermal stress resilience [19]. This name directly identifies the primary role of these integral subcellular structures: maintaining cellular homeostasis and stability against environmental stress, much like a “biological damper” to confer resilience. Under this framework, previously described “polyphosphate granules” and “acidocalcisomes” can be considered as species-specific manifestations of the stabilisomes.

Beyond fundamental research, the potential for practical applications, particularly in biomedicine, is a significant driver for the development of effective purification processes for stabilisomes [18]. For instance, the reported anti-inflammatory effects of biogenic polyphosphate nanogranules from other cyanobacteria [20], underscoring the potential of stabilisomes as a promising natural biomaterial. Despite their functional significance, isolating stabilisomes with high purity and preserved integrity from cyanobacteria is a major technical challenge in bioseparation. Conventional methods such as density gradient centrifugation or gel filtration often suffer from cytoplasmic contamination, low yield, or structural degradation [21,22,23,24]. Furthermore, the robust cell walls of thermophilic cyanobacteria make cell disruption without organelle damage particularly difficult [25,26,27]. Overcoming these technical barriers is essential for enabling detailed biochemical and structural studies of stabilisomes and exploring their biotechnological potential.

Here, we establish and validate a high-purity purification strategy for isolating intact stabilisomes from thermophilic cyanobacteria. To achieve this, we developed a multi-step protocol integrating gentle sonication, differential centrifugation, ultrafiltration, iodixanol density gradients, and a detergent-assisted surface wash. We then conducted a comprehensive validation of the purified stabilisomes using a multi-dimensional approach, including transmission electron microscopy (TEM), energy-dispersive X-ray spectroscopy (EDS), and nanoparticle tracking analysis (NTA). This study aimed to achieve two primary objectives: First, to provide the ultimate biochemical validation of the method’s high-resolution; and second, to leverage this validated method to elucidate, for the first time, the core chemical composition of stabilisomes. The core of this work is to establish a robust methodological platform that enables the in-depth analysis of these critical organelles, unveiling the chemical basis of cyanobacterial thermal resilience.

## 2. Materials and Methods

### 2.1. Thermophilic Cyanobacterial Strains and Culture Conditions

To investigate the ubiquity of stabilisomes in thermophilic cyanobacteria and to validate the universality of our newly established isolation method, we selected seven thermophilic cyanobacterial samples from geographically and thermally distinct hot springs across China (Figure S1, Table S1). To ensure broad representation, these isolates encompassed a significant phylogenetic variability, including strains of *Synechococcus* sp., *Nostoc* sp., and *Lyngbya* sp., and notably included two stable mixed cultures (GDSG-3 and GDSG-4) to mimic more natural consortia. Among these, the representative strain FJSJ-1 (*Synechococcus* sp.) was selected for in-depth multi-omics analyses, while the remaining isolates were used for subsequent comparative quantitative studies. Each strain was cultivated in BG11 medium at its respective native temperature (Table S1), under a 12 h:12 h light–dark cycle at an intensity of photosynthetic photon flux density (PPFD) of approximately 40 ± 15 μmol m^−2^ s^−1^ (converted from 3000 ± 1000 lx for cool white fluorescent lamps [Nanjing Lisigao Electronic Technology Co., Ltd., Nanjing, China] ). Cultures were harvested during the late exponential to early stationary phase (14–28 days) for stabilisomes extraction. All experiments were performed using three independent biological replicates (*n* = 3).

### 2.2. Comparative Assessment of Purification Strategies

**Figure 1:**
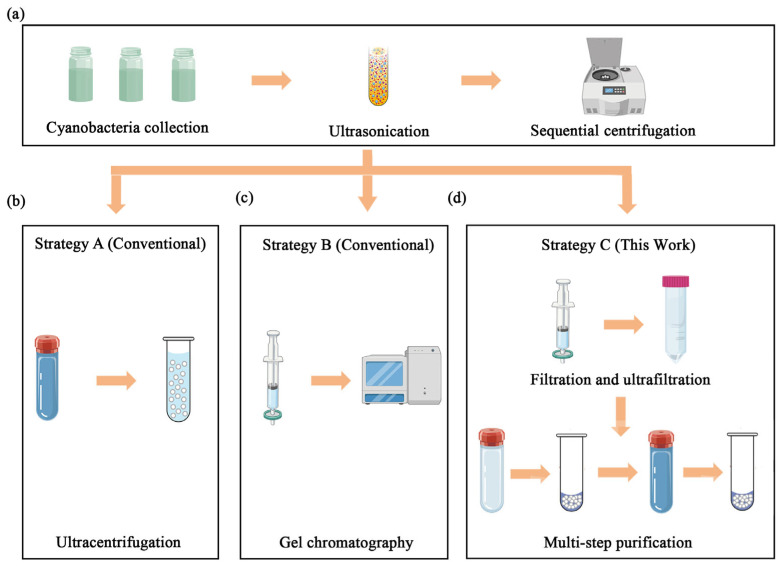
Schematic comparison of three purification strategies for stabilisomes isolation. The diagram illustrates the key steps involved in the different methods evaluated. (**a**) The initial harvesting and pre-purification steps common to all workflows, including cell collection, washing, and initial differential centrifugation to remove large debris. (**b**) The single-step ultracentrifugation strategy (strategy A). (**c**) The gel exclusion chromatography strategy (strategy B). (**d**) The multi-step combined strategy developed in this study, which synergistically integrates optimized cell lysis, ultrafiltration, density gradient centrifugation, and a final detergent-assisted surface wash (strategy C).

To identify the optimal method for stabilisome isolation, three distinct purification strategies were comparatively assessed in parallel (Figure 1). We established a standardized workflow where initial cell disruption and debris removal steps were identical for all three protocols (Figure 1a), ensuring that any differences in yield or purity could be attributed solely to the separation method itself.

#### 2.2.1. Initial Cell Lysis and Clarification

Cells were harvested by centrifugation at 6000× *g* for 10 min, washed four times with cold 0.15 M NaCl to remove extracellular impurities, and resuspended in isotonic NaCl. The suspensions were subjected to ultrasonic disruption using a probe sonicator (Nanjing Xianou Instruments Manufacture Co., Ltd., Nanjing, China) (50% amplitude, 20 kHz frequency) for 5 min in an ice bath (a pulse cycle consisting of a 5 s on-period followed by a 5 s off-period). The lysate was sequentially centrifuged at 300× *g*, 2000× *g*, and 10,000× *g* for 10 min each at 4 °C to remove intact cells and large debris (Figure 1a). The resulting clarified supernatant was examined microscopically to verify successful cell lysis and stabilisomes release before being used as the starting material for the three parallel purification strategies.

#### 2.2.2. Strategy A: Single-Step Ultracentrifugation

The clarified supernatant from step Section 2.2.1 was subjected to a single ultracentrifugation step at 150,000× *g* for 1 h at 4 °C using an Optima XPN-100 ultracentrifuge (Beckman Coulter, Brea, CA, USA), following the classical protocol described by the resulting pellet, serving as the conventional control group, expected to contain stabilisomes and other dense cellular components, was then resuspended in NaCl buffer for analysis (Figure 1b).

#### 2.2.3. Strategy B: Gel Exclusion Chromatography

The clarified supernatant from step Section 2.2.1 was concentrated using a 100 kDa molecular weight cut-off (MWCO) ultrafiltration membrane (Nanjing Banma Experimental Equipment Co., Ltd., Nanjing, China) and then loaded onto a Sephacryl S-100 size-exclusion column (Cytiva, Marlborough, MA, USA), pre-equilibrated with NaCl buffer (100 mM, pH 7.2–7.4). This chromatography strategy was adapted from established protocols for organelle separation. Fractions were collected and the ones corresponding to the void volume, where large particles like stabilisomes are expected to elute, were pooled for analysis [20] (Figure 1c).

#### 2.2.4. Strategy C: Optimized Multi-Step Protocol

The clarified supernatant from step Section 2.2.1 was first passed through 0.45 μm and 0.22 μm (Nanjing Banma Experimental Equipment Co., Ltd., Nanjing, China) syringe filters to remove residual debris. Concentration was achieved via a 100 kDa MWCO ultrafiltration spin device (Nanjing Banma Experimental Equipment Co., Ltd., Nanjing, China), and the concentrate was then pelleted by ultracentrifugation at 150,000× *g* at 4 °C for 1 h. The resulting pellet was resuspended in NaCl buffer (100 mM, pH 7.2–7.4) and carefully layered onto a pre-formed 12-step iodixanol gradient (OptiDensity™, Nanjing Jiyi Biotechnology Co., Ltd., Nanjing, China). After a second ultracentrifugation using an Optima XPN-100 instrument (Beckman Coulter, Brea, CA, USA) at 150,000× *g* for 1 h at 4 °C, the band located at the 10% iodixanol interface was carefully collected by pipetting from the top. To remove adsorbed cytoplasmic proteins, the collected band was incubated with 0.3% Tween-20 (Nanjing Banma Experimental Equipment Co., Ltd., Nanjing, China) at room temperature with gentle agitation on a rocker for 15 min. The sample was then washed three times with NaCl buffer using the same 100 kDa ultrafiltration device (each wash performed by centrifuging at 4000× *g* for 10 min). The final purified stabilisomes fraction was resuspended in a minimal volume of NaCl buffer and stored at −80 °C until further characterization (Figure 1d).

### 2.3. Transmission Electron Microscopy (TEM) and Energy-Dispersive X-Ray Spectroscopy (EDS)

Purified stabilisomes were fixed in 2.5% glutaraldehyde (0.1 M phosphate buffer, pH 7.2) for 2 h, rinsed, and post-fixed in 1% osmium tetroxide for 1 h. Samples were dehydrated in a graded ethanol series, infiltrated with epoxy resin, and polymerized at 60 °C. Ultrathin sections (70–90 nm) were cut using an ultramicrotome (Leica EM UC7, Leica Microsystems, Vienna, Austria) and mounted on copper grids. Sections were stained with uranyl acetate and lead citrate and imaged with a Talos F200X G2 TEM (Thermo Fisher Scientific, Waltham, MA, USA). Elemental composition was assessed using EDS on selected regions.

### 2.4. Nanogranule Tracking Analysis (NTA)

Size and concentration of stabilisomes were determined using a NanoSight NS300 instrument (Malvern Panalytical, Malvern, UK). Samples were diluted to 1 × 10^7^ to 1 × 10^9^ granules/mL using ultrapure water. Five replicate 60 s videos were recorded and analyzed using NTA software (v3.4) to obtain mean hydrodynamic diameter and granule concentration.

### 2.5. Sample Preparation for Liquid Chromatography–Tandem Mass Spectrometry (LC-MS/MS)

Sample preparation and subsequent LC-MS/MS analyses were conducted by Wuhan Metware Biotechnology Co., Ltd. (Wuhan, China).

#### 2.5.1. Proteomic Sample Preparation

Stabilisomes were lysed in SDT buffer (4% SDS, 100 mM Tris-HCl, 1 mM DTT, pH 7.6), sonicated, and heated to denature proteins. Lysates were centrifuged to remove debris, and supernatants were alkylated with iodoacetamide. Proteins were precipitated with four volumes of pre-chilled acetone and washed, then re-dissolved in 8 M urea buffer. Proteins were digested with trypsin (Promega, Madison, WI, USA) overnight at 37 °C, desalted using C18 resin columns (Shimadzu Corporation, Kyoto, Japan), and stored for LC-MS/MS analysis.

#### 2.5.2. Metabolite Extraction

For untargeted metabolomics, 200 μL of purified stabilisome suspension was mixed with 200 μL of cold 70% methanol containing internal standards. The mixture was vortexed for 10 min, centrifuged (10,000× *g*, 3 min, 4 °C), and filtered through a 0.22 μm membrane. The filtrate was transferred into autosampler vials for analysis.

### 2.6. Proteomic and Metabolomic Analyses by LC-MS/MS

#### 2.6.1. Proteomics Analyses

Peptides were separated by a Vanquish Neo Ultra-high-performance liquid chromatography (UHPLC) system (Thermo Fisher) on a PepMap Neo column and analyzed on an Orbitrap Astral mass spectrometer (Thermo Fisher Scientific, Waltham, MA, USA) in data-independent acquisition (DIA) mode. Spectra were acquired across 299 isolation windows, processed using DIA-NN (v1.8.1), and searched against the *Thermosynechococcus elongatus* Uniprot database. The false discovery rate was controlled at <1%.

#### 2.6.2. Metabolomics Analyses

Metabolite separation was conducted on a Shimadzu LC-30A UHPLC system equipped with a Waters ACQUITY Premier HSS T3 column (1.8 µm, 2.1 mm × 100 mm; Waters Corporation, Milford, MA, USA) at 40 °C. A linear gradient of 0.1% formic acid in water (A) and 0.1% formic acid in acetonitrile (B) was applied at a flow rate of 0.4 mL/min, with an injection volume of 4 µL. The gradient program was: 0–2 min, 5–20% B; 2–6 min, 20–99% B; 6–7.5 min, 99% B; 7.5–10 min, re-equilibration at 5% B. The separated metabolites were analyzed on a SCIEX TripleTOF 6600+ mass spectrometer (SCIEX, Framingham, MA, USA). based on rigorous matching of retention times and MS/MS fragmentation patterns. Representative spectral mirror plots confirming the structural identification of key metabolites (e.g., L-Phenylalanine, citric acid, adenosine, and spermine) are provided in Figure S2.

Quality Control (QC) and data processing to ensure data reliability, pooled QC samples were prepared by mixing equal aliquots of all experimental samples and were injected after every 10 experimental samples to monitor instrument stability. Blank samples were analyzed to detect potential carryover. Raw data were processed using ProteoWizard (v3.0) and XCMS for peak alignment. To correct for signal drift and technical variation, the Support Vector Regression (SVR) normalization method was applied to the peak area data. Only metabolites with a Coefficient of Variation (CV) < 0.5 in QC samples were retained for subsequent analysis, ensuring high data reproducibility (CV < 0.5 for >85% of metabolites in QC samples).

### 2.7. Biochemical Quantification of Stabilisome Components

#### 2.7.1. Dry Weight Measurement

Aliquots of stabilisome suspensions were freeze-dried using a vacuum lyophilizer (Shanghai Jingxin Industrial Development Co., Ltd., Shanghai, China). Samples were weighed before and after drying using a microbalance (0.001 mg resolution). The dry weight was calculated and used as the basis for percentage composition calculations.

#### 2.7.2. Component Quantification

Dried stabilisomes were lysed and homogenized in appropriate buffers. Component concentrations were then measured using specific commercially available kits. Total protein content was determined using a bicinchoninic acid (BCA) assay kit (Coolaber, Nanjing, China). Triglyceride (TG) content was measured with an enzymatic kit (Yuanye Bio-Technology, Shanghai, China). The contents of phosphatidylethanolamine (PE) and phosphatidylglycerol (PG) were quantified using microbial ELISA kits (Jiaoziteng, Shanghai, China). The contents of free amino acids and alkaloids were determined using their respective ELISA kits (Macklin, Shanghai, China). Polyamine content was quantified using a plant polyamine ELISA kit (Ruifan Bio-Technology, Shanghai, China). The concentrations of key nucleotides, including ATP, ADP, and AMP, were quantified by High-Performance Liquid Chromatography (HPLC) using a detection kit from Solarbio (Beijing, China). All assays followed the manufacturers’ instructions, and data were normalized to the stabilisome dry weight.

For all commercial assay kits, calibration was strictly performed using the external standards provided by the manufacturers, generating linear standard curves with R^2^ > 0.99 within the detection range. To minimize potential matrix effects caused by extraction buffers, stabilisome samples were diluted at least 10-fold with the assay buffer prior to measurement, ensuring the analyte concentrations fell within the linear range of the standard curve.

#### 2.7.3. Quantification of polyP

The polyP content in purified stabilisomes was quantified using a persulfate digestion-molybdenum blue colorimetric assay. Briefly, a known dry weight of lyophilized stabilisomes was resuspended in ultrapure water. The suspension was then digested with potassium persulfate (K_2_S_2_O_8_) at a final concentration of approximately 0.8% (*w*/*v*) at 121 °C for 60 min to convert all phosphorus forms to orthophosphate (Pi), which was then quantified using the molybdenum blue method [28].

### 2.8. Quantification of Metal Ion Content

The metal ion content in stabilisomes was quantified using Inductively Coupled Plasma Mass Spectrometry (ICP-MS) (Agilent 7850, Agilent Technologies, Santa Clara, CA, USA). Briefly, an accurately weighed aliquot (~100 mg) of the lyophilized stabilisome powder was subjected to microwave-assisted acid digestion with nitric acid until a clear solution was obtained. The digestate was then diluted to a suitable concentration with ultrapure water, filtered through a 0.45 μm membrane, and analyzed by ICP-MS to determine the concentrations of the major metal cations of Ca^2+^, Mg^2+^, K^+^, and Na^+^. The final results were normalized to the initial dry weight of the sample.

### 2.9. Statistical Analysis

Data are presented as mean ± standard deviation (SD). Statistical comparisons were performed using one-way analysis of variance (ANOVA) or Student’s *t*-tests as appropriate, with significance defined as *p* < 0.05. All graphs were generated using OriginPro 2018 (OriginLab Corporation, Northampton, MA, USA) and GraphPad Prism 9 (GraphPad Software, San Diego, CA, USA).

## 3. Results and Discussion

### 3.1. Establishment and Physical Validation of a Purification Method for Thermophilic Cyanobacterial Stabilisomes

To isolate stabilisomes from thermophilic cyanobacteria with both high purity and integrity, we designed and executed a stepwise experimental workflow that incorporates multi-dimensional validation. This chapter will first detail the establishment, optimization, and physicochemical validation of our novel purification method.

#### 3.1.1. Initial Identification of Stabilisomes

**Figure 2:**
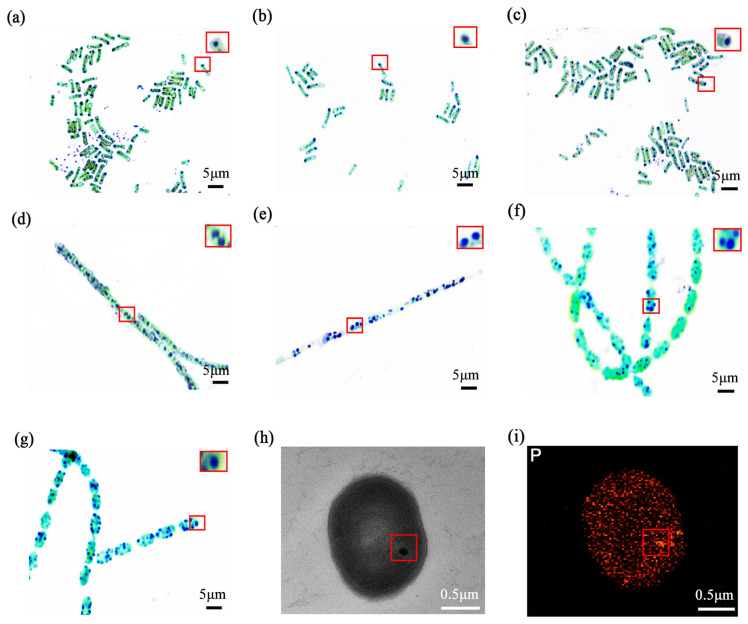
The optics and electron microscopy images of thermophilic cyanobacterial stabilisomes. Metachromatic staining of seven isolates: FJSJ-1 (**a**), GDSG-1 (**b**), GDSG-2 (**c**), JLCBS-1 (**d**), GDMZ-1 (**e**), GDSG-3 (**f**), and GDSG-4 (**g**). The red square in (**a**) indicates the region magnified in the inset, which shows a magnified view of typical stained stabilisomes. Scale bar = 5 μm. Transmission electron microscopy (TEM) images reveal electron-dense spherical stabilisomes in the cytoplasm (**h**), Energy dispersive X-ray spectroscopy (EDS) analysis of the stabilisomes, showing that it is enriched in the element phosphorus (**i**). Scale bar = 0.5 μm.

The thermophilic cyanobacterial isolates at 7 hot spring sampling sites used in this study were selected to represent a broad morphological and phylogenetic variability (Table S1). They encompassed four key genera, specifically including three distinct strains of *Synechococcus* sp., one strain of *Nostoc* sp., one strain of *Lyngbya* sp., and two stable mixed cultures containing *Anabaena* sp and *Synechococcus* sp. Using heterochromatic granule staining, we observed dense blue-violet intracellular inclusions in all selected strains (Figure 2a–g). A magnified view of typical stained stabilisomes, highlighted within a red box, further illustrates these characteristic granules.

For detailed ultrastructural analysis, the *Lyngbya* sp. (GDMZ-1) strain was selected because its stabilisomes were observed to occupy a proportionally larger intracellular volume, making it an ideal target for high-resolution TEM imaging. TEM revealed spherical, electron-dense granules (~100–200 nm) dispersed within the cytoplasm of this strain (Figure 2h), while EDS showed strong enrichment in phosphorus (Figure 2i), suggesting the presence of typical polyP-rich granules consistent with stabilisomes. These features are highly consistent with descriptions of polyP granules in various prokaryotes, suggesting that stabilisomes are common, phosphorus-rich subcellular structures in cyanobacteria [14,15,29].

#### 3.1.2. Construction of a Multi-Step Purification Protocol

**Figure 3:**
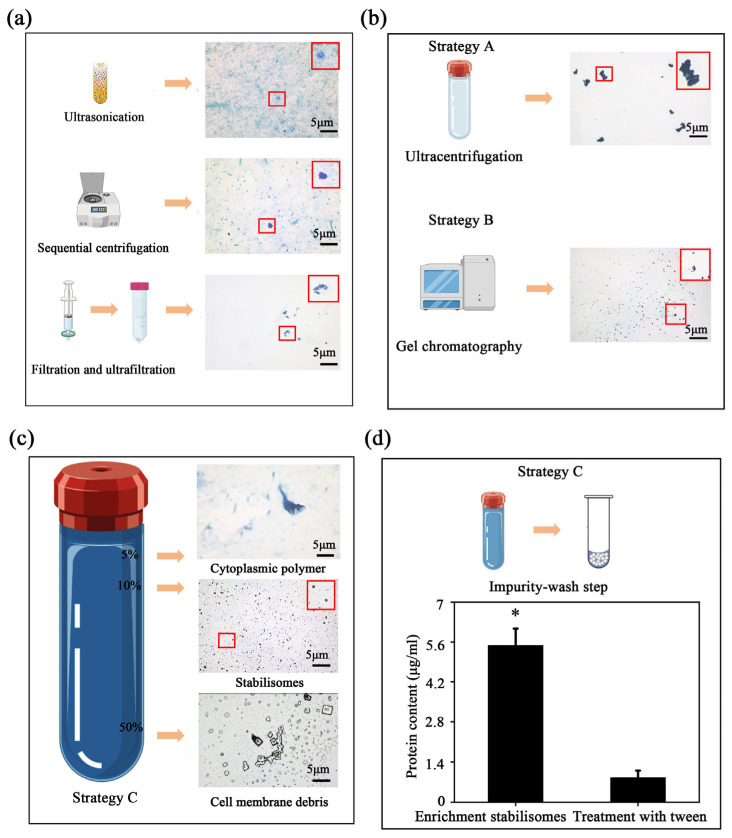
Optimization and validation of the multi-step purification protocol. In the schematic illustrations, the blue-violet balls represent stabilisomes, while the blue-green balls represent cellular impurities. Metachromatic staining showing the gradual enrichment of stabilisomes after the initial processing steps; the red box provides a magnified view of the granules, with the arrow highlighting typical stabilisomes (**a**). Limitations of conventional methods: The upper panel shows granule aggregation and impurity co-precipitation from single-step ultracentrifugation (Strategy A); the middle panel shows the inadequate separation by gel exclusion chromatography (Strategy B) (**b**). After the core purification step of iodixanol density gradient centrifugation, stabilisomes form a clear, distinct stained band at the 10% (*w*/*v*) concentration interface (**c**). Validation of the final wash step: The bar chart compares the total protein content before and after the 0.3% Tween-20 wash, showing a significant reduction and demonstrating its effectiveness in removing adsorbed surface proteins (Strategy C) (**d**). Data are presented as mean ± SD (*n* = 3 independent biological replicates). * *p* < 0.05. Scale bars = 5 μm.

To overcome isolation challenges, we evaluated three strategies (Strategies A–C, detailed in Section 2.2 and Figure 1). While Strategies A and B resulted in significant aggregation or impurity co-precipitation, the optimized Strategy C successfully achieved high purity and structural integrity. Given that stabilisomes exhibit highly conserved structural characteristics across the seven diverse thermophilic cyanobacteria studied here, *Synechococcus* sp. was selected as the most representative model organism for in-depth characterization. This strain was chosen not only for its ubiquity in geothermal environments but also for its challenging small, unicellular morphology, which provides a stringent test for a new isolation method. To further evaluate the method’s transferability beyond this representative model, we subsequently applied the optimized protocol to the other phylogenetically diverse isolates, thereby validating its broad applicability. This protocol integrates three core innovations (Figure 1c). First, for cell lysis, we employed an optimized sonication protocol instead of conventional boiling or enzymatic methods. This choice was critical to balance effective cell disruption with the preservation of stabilisome integrity, thereby avoiding known artifacts like polyP co-precipitation that can occur with other techniques in thermophiles [30]. Second, the lysate was subjected to differential centrifugation and 100 kDa ultrafiltration to remove large cellular debris and background macromolecules, resulting in an initial enrichment of target granules (Figure 3a).

Notably, simple gel exclusion chromatography alone is insufficient to separate stabilisomes from similarly sized protein complexes such as phycobilisomes, due to overlap in hydrodynamic profiles and molecular mass ranges [31,32]. To address this limitation, we implemented iodixanol density gradient ultracentrifugation using a 12-step discontinuous gradient with concentrations ranging from 0% to 60% (*w*/*v*) (Figure 3c). The iso-osmotic nature of iodixanol allows for gentle separation of intact subcellular structures, avoiding the hyperosmotic damage typically associated with sucrose-based media [33,34]. Stabilisomes consistently localized at the 10% interface (Figure 3c), indicating precise resolution under iso-density conditions.

A final surface purification step using low-concentration non-ionic detergent (Tween-20) was introduced to remove non-specifically adsorbed cytoplasmic proteins. This strategy, previously applied in virus purification workflows [35], is here employed for the first time in cyanobacterial organelle isolation and markedly improves final sample purity (Figure 3d). Throughout the protocol, each processing stage was validated by heterochromatic granule staining observations to monitor stabilisome enrichment and integrity (Figure 3).

The physicochemical characteristics of the purified stabilisomes (Figure 3c)—namely their consistent morphology and strong positive reaction to metachromatic staining—closely resemble those of acidocalcisomes and previously characterized polyP-rich granules [36,37]. These results collectively suggest that our stepwise methodological refinements represent a reliable, structure-preserving, and biochemically faithful strategy for the isolation of intact stabilisomes from thermophilic cyanobacteria.

#### 3.1.3. Comparative Analysis of Different Purification Strategies

To comprehensively assess the superiority of our newly constructed protocol, we first performed a qualitative comparison of the products via metachromatic staining, followed by a quantitative benchmark against two conventional strategies: single-step ultracentrifugation (Figure 1a) and gel exclusion chromatography (Figure 1b). Comparative analysis of the products revealed the limitations of conventional methods. The ultracentrifugation product exhibited significant granule aggregation (Figure 3b), a phenomenon known to cause the non-specific co-precipitation of cytoplasmic impurities [31]. In contrast, while the gel chromatography product showed better granule integrity (Figure 3b), the method is inherently limited in its ability to resolve stabilisomes from other co-eluting macromolecular complexes of similar size, such as phycobilisomes [32]. In contrast, the multi-step workflow yielded visibly purer and morphologically uniform granules, suggesting superior separation performance (Figure 3c).

To quantitatively evaluate the efficiency of each method, we adopted the granule-to-protein ratio as a key metric for purity [38]. We know that stabilisomes and exosomes—for which this metric was established as a standard [39]—are fundamentally different in chemical composition and density [40], and therefore, their absolute ratio values are not directly comparable. However, the core technical challenge is identical in both purification problems: the effective separation of a target nanogranule from a vast excess of soluble, contaminating cytoplasmic proteins, a major focus of purity assessment in the field [41]. Thus, the granule-to-protein ratio is employed here not as an absolute measure of purity across organelle types, but as a highly effective internal and relative metric to benchmark the performance of different purification strategies at this specific task [41].

The results revealed a dramatic enhancement with our multi-step protocol (Table 1). The final workflow yielded a granule-to-protein ratio of 1.06 × 10^7^ granules/μg, which is approximately 300-fold higher than strategy B (3.56 × 10^4^ granules/μg) and an unprecedented 10,000-fold higher than strategy A (9.95 × 10^2^ granules/μg). This vast, orders-of-magnitude improvement provides unequivocal evidence of our method’s superior ability to remove co-purifying protein contaminants.

#### 3.1.4. Physicochemical Characterization of Purified Stabilisomes

**Figure 4:**
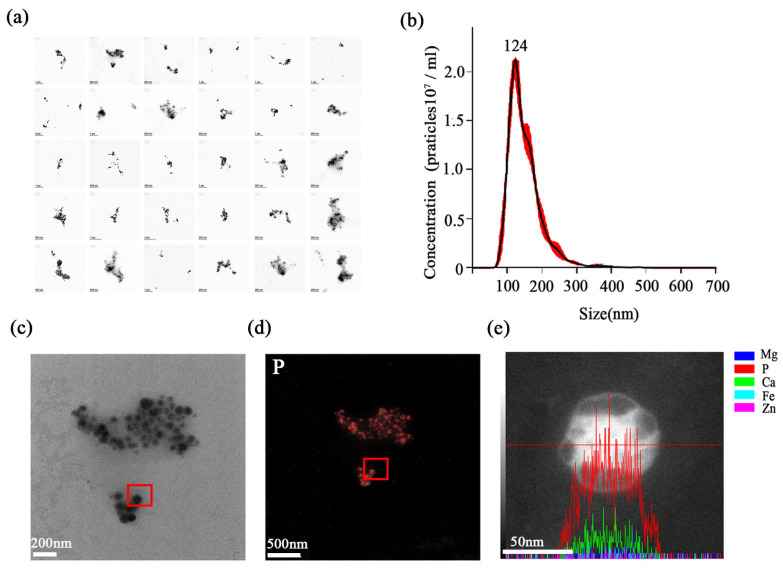
Physicochemical characterization of purified stabilisomes. A representative TEM image from a survey of 30 random fields, showing a dense population of purified stabilisomes closely packed together while maintaining their individual structural integrity (**a**). Nanoparticle Tracking Analysis (NTA) confirming the narrow size distribution with a mean hydrodynamic diameter of ~100–200 nm; the red dots represent the raw data points, and the black curve indicates the fitted distribution (**b**). This panel illustrates the selection of a single, representative stabilisome for the subsequent elemental analyses (**c**) shown in (**d**,**e**). Energy-Dispersive X-ray Spectroscopy (EDS) mapping of the granule selected in (**c**), revealing strong phosphorus enrichment (**d**). EDS line-scan analysis across the same granule, confirming its enrichment in both phosphorus (P) and calcium (Ca), where the red line indicates the path of the EDS line-scan (**e**). Scale bars were 200 nm (**a**,**c**), 500 nm (**d**) and 50 nm (**e**).

The final product was assessed through a suite of structural and compositional analyses. A survey of 30 randomly selected fields by TEM revealed a dense population of stabilisomes that were closely packed yet remained as discrete, structurally intact granules (Figure 4a). Nanogranule tracking analysis (NTA) revealed a narrow size distribution centered around 100–200 nm (Figure 4b). Building on this, a representative granule was then selected for detailed elemental analysis (Figure 4c). EDS mapping and line-scan confirmed that the granules retained strong enrichment in P and calcium (Ca) (Figure 4d,e), in agreement with known polyP storage granules. This element composition is highly consistent with the reported chemical fingerprints of acidic calcium or polyP granules [36,37]. This result affirms the specificity of our method for isolating stabilisomes rather than general membrane vesicles. In summary, we have developed and validated a reproducible, robust method for the isolation of stabilisomes from thermophilic cyanobacteria.

### 3.2. Biochemical Characterization: Assessment of Structural Integrity and Elucidation of the Stabilisome Chemical Composition

While the previous section provided the physical validation for our protocol, a truly robust isolation method requires final biochemical validation to substantiate its high structural integrity. This chapter therefore aims to provide this ultimate validation through in-depth multi-omics and quantitative analyses, and in doing so, elucidate for the first time the core chemical composition of the stabilisomes and the principles governing its makeup across different thermophilic cyanobacteria. To achieve this dual objective, we first performed in-depth proteomic and metabolomic analyses on the representative strain, *synechococcus* sp., to identify its full complement of molecular components. Subsequently, we performed a comparative quantitative analysis across all seven thermophilic cyanobacterial samples to establish the conserved compositional framework and the extent of inter-strain variations in component abundance.

#### 3.2.1. Proteomic Validation: Confirming the Authenticity and Integrity of the Isolate

Comprehensive LC-MS/MS analysis identified 937 non-redundant proteins within purified stabilisome fractions, representing a highly complex diverse proteome (Table S2). Among them were key enzymes involved in phosphate metabolism and stress response, such as polyphosphate kinase (PPK), ATP synthase subunit a, phosphate-binding proteins, histidine kinase, and agmatine deiminase (Table 2).

This detailed proteomic profile provides compelling biochemical evidence for the superiority of our isolation method from two key perspectives: specificity and structural preservation. The method exhibits high specificity, favoring the capture of authentic stabilisomes rather than non-specific precipitates of cytosolic proteins. A central challenge in subcellular proteomics is distinguishing authentic organelle-localized proteins from co-purified cytosolic contaminants [31]. It is important to note that due to the high cellular abundance of phycobilisomes and thylakoid membranes in cyanobacteria, peptides associated with these structures were co-identified. However, unlike non-specific precipitation, the stabilisome fraction showed a specific enrichment of the core marker PPK and associated transporters, confirming the selectivity of the isolation procedure despite the background of abundant cellular proteins. A non-specific method would be expected to yield a product dominated by a random assortment of the most abundant cellular proteins. Our results, however, revealed a network dominated by a set of biochemically related core proteins. Crucially, this core protein composition is highly consistent with that established for similar polyP-storing organelles through extensive research in the field, including cornerstone enzymes like PPK and ATP synthase [45,46]. This finding, corroborated by the extremely high granule-to-protein ratio achieved, demonstrates our method’s ability to overcome the co-purification bottleneck and specifically enrich for the target organelle.

Moreover, the successful retention of specific protein types directly reflects the structure-preserving nature of our protocol. Crucially, we successfully retained large, multi-subunit complexes like ATP synthase and conformation-sensitive signaling proteins like histidine kinase [47]. It is well-established that harsh separation conditions can easily lead to the dissociation or inactivation of such fragile protein assemblies, rendering them undetectable in final proteomic analyses [48,49,50]. Therefore, the systematic identification of these “delicate” proteins serves provides strong evidence that our method is gentle enough to preserve the native protein assemblies of the stabilisomes.

In terms of the purification strategy, the inclusion of a mild 0.3% Tween-20 wash step was a strategic trade-off to ensure high purity. Tween-20 is a non-ionic surfactant that is widely recognised for its gentle properties. It has been demonstrated that this allows for the solubilisation of peripheral contaminants without disrupting the lipid bilayer or stripping integral membrane proteins at low concentrations [51]. It is acknowledged that the possibility of minimal loss of surface-associated lipids or loosely bound peripheral proteins during this step cannot be strictly excluded. However, a plethora of lines of evidence support the overall structural integrity of the isolated stabilisomes. Firstly, complex transmembrane machineries such as ATP synthase have been retained (Table 2). Secondly, enclosed vesicular morphology has been observed via TEM (Figure 4). Collectively, these data suggest that the 0.3% Tween-20 treatment effectively removed cytosolic impurities while leaving the core organelle architecture intact.

Building on the observed authenticity and integrity of this proteome, we can further leverage its composition to infer the biological functions of the stabilisomes. First, the co-enrichment of numerous enzymes related to energy conversion and phosphate metabolism, such as ATP synthase and PPK, suggests that the stabilisomes are a key hub for cellular energy and phosphate homeostasis [12,41]. Second, the presence of various signal transduction proteins, including histidine kinases, suggests that the stabilisomes are not a passive storage unit but an active, dynamic center for sensing and responding to environmental stress [52]. Finally, the identification of specific amino acid metabolic enzymes, such as arginine deiminase, points towards specialized metabolic pathways, potentially for the synthesis of protective compounds under stress [53]. In summary, this robust proteome provides the first solid molecular evidence to define the stabilisomes as a multifunctional organelle that integrates energy conversion, stress sensing, and responsive metabolism.

#### 3.2.2. Untargeted Metabolomics Reveals a Chemically Structured, Enclosed Microenvironment

**Figure 5:**
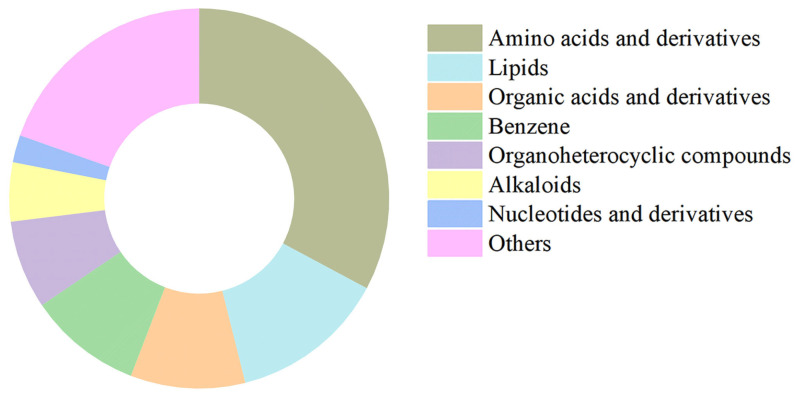
Chemical classification of metabolites identified in purified stabilisomes from the representative *Synechococcus* sp. The chart displays the classification of 1076 metabolites identified from purified stabilisomes of the representative *Synechococcus* sp. Percentages represent the proportion of the number of metabolite types within each class relative to the total number of identified types. The results clearly show that amino acids and their derivatives (33%) represent the most diverse category by a significant margin, followed by lipids (13%), organic acids and derivatives (10%), and benzene and substituted derivatives (10%). Data are presented as mean ± SD (*n* = 3 independent biological replicates).

To further assess the high structural integrity of our isolation method and to analyze the metabolite composition of the purified stabilisomes, we performed untargeted LC-MS metabolomics on the purified stabilisomes. After a rigorous purification process designed to completely remove external free small molecules, we still successfully identified 1076 metabolites from within the stabilisomes (Table S3). Classification of these compounds revealed high chemical variability (Figure 5), with amino acids and derivatives accounting for 33% of all compound types, followed by lipids (13%), organic acids (10%), and benzene derivatives (10%).

These rich and biochemically complex metabolomes provide robust, biochemical-level evidence for structural and functional integrity of our isolation method. The key validation lies not just in the quantity of metabolites retained, but in the preservation of the co-existence of specific enzymes with their corresponding substrates and products. We observed the preservation of several such enzyme-metabolite sets. For instance, arginine decarboxylase was identified alongside its substrate arginine and product agmatine. The composition of this complete set of biochemical components is highly consistent with the polyamine biosynthesis pathway previously characterized in the model cyanobacterium *Synechocystis* sp. PCC 6803 [54,55], which supports the robust of our product. While enzymatic activities were not directly assayed in this study, this co-localization of an enzyme with its substrate and product in a confined space suggests a potential for efficient biochemical reactions, a phenomenon conceptually similar to “metabolic channeling” [56]. This strategy is common in other key organelles; for instance, enzymes of the tricarboxylic acid (TCA) cycle in mitochondria form supermolecular complexes to accelerate metabolic flux [57]. Therefore, the ability to preserve the co-localization of specific enzymes with their substrates and products is the most direct evidence for the structure-preserving of our isolation method.

Furthermore, the abundance of positively charged amino acids and organic acids suggests electrostatic balancing of the highly anionic polyP core, recapitulating the acidocalcisome-like features previously described [58,59]. This feature is highly conserved evolutionarily; for example, in the unicellular green alga *Chlamydomonas reinhardtii* [60], its polyP-rich acidic vacuoles have been described to be functionally analogous to acidocalcisomes, similarly participating in ion storage and osmoregulation. The ability to clearly reproduce this ancient chemical signature in our thermophilic cyanobacterial samples not only supports the biological authenticity of our product but also highlights our method’s capacity to preserve the complex, structured biochemistry of the stabilisomes.

Building on the high-confidence authenticity and integrity of metabolomes, its unique composition provides the first key insights into the potential biological functions of the stabilisomes. First, the overwhelming dominance of amino acids and their derivatives suggests that the stabilisomes are key hub for amino acid storage and metabolism, potentially providing a rapid resource buffer under nutrient limitation or stress [61]. Second, the co-retention of key energy currency molecules like ATP and ADP with various organic acids implies a potential role in local energy metabolism and cellular energy homeostasis [62]. Finally, the identification of various bioactive small molecules, such as the polyamine agmatine, points towards specialized metabolic functions in synthesizing protective compounds against environmental stressors like oxidation or high temperature [63]. In summary, the metabolomic analysis not only provides compelling evidence that our isolation protocol is structure-preserving, but also suggests for the first time the vast potential of the stabilisomes as a highly dynamic, multifunctional biochemical center that integrates nutrient storage, energy buffering, and stress-responsive metabolism.

#### 3.2.3. Targeted Quantification Delineates a Conserved Chemical Architecture

**Figure 6:**
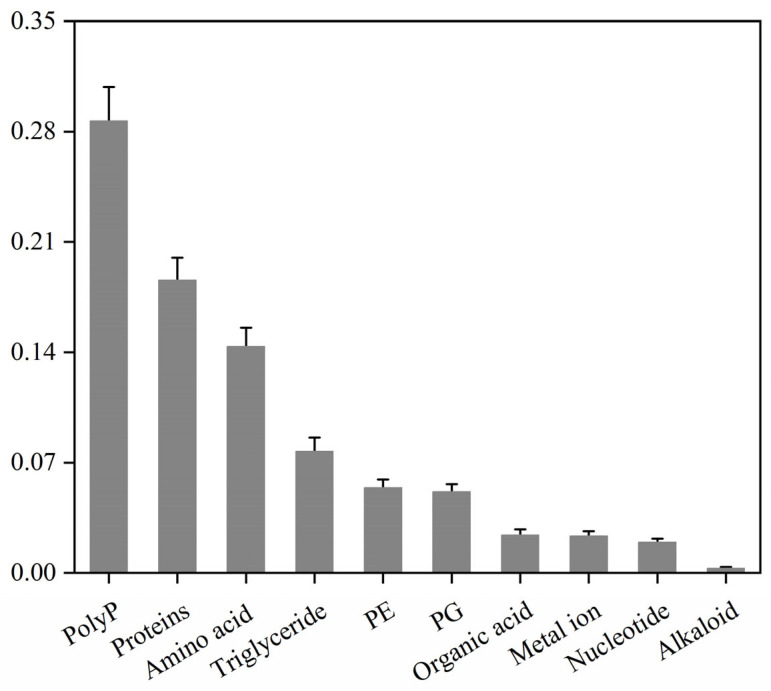
Mass percentage compositions of stabilisome components from the representative *Synechococcus* sp. The chart displays the mass proportion of polyP, protein, amino acids, lipids, and other major components from purified stabilisomes of a single representative species. All values were determined by specific quantitative assays and are expressed as a percentage of the total sample dry weight. Data are presented as mean ± SD (*n* = 3 independent biological replicates).

While the preceding multi-omics analyses revealed the basic components of the stabilisomes, such high-throughput techniques have inherent limitations in absolute quantification. Therefore, to more precisely and definitively define the core chemical makeup of the stabilisomes, we applied a suite of standardized biochemical assays for absolute quantification. Analysis of stabilisomes from the representative strain, *Synechococcus* sp. (Table S1), defined its detailed chemical compositions, which were found to comprise polyP (29%), protein (19%), amino acids (14%), triglycerides (8%), phosphatidylethanolamine (PE) (5%), phosphatidylglycerol (PG) (5%), organic acids (2%), metal ions (2%), and nucleotides (2%), among other trace components (Figure 6).

**Figure 7:**
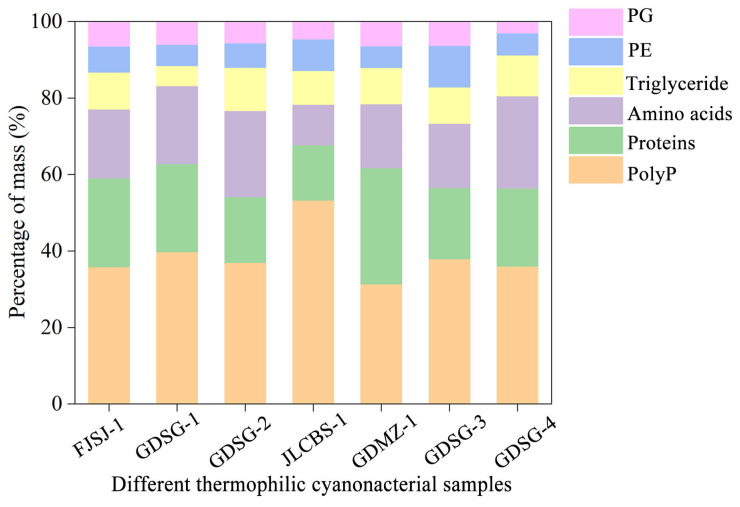
Comparison of mass percentages of major biochemical components in stabilisomes from seven thermophilic cyanobacterial samples. The chart displays the mass percentages of polyP, protein, amino acids, triglycerides, phosphatidylglycerol (PG), and phosphatidylethanolamine (PE) for each species. Each strain is identified by its unique Strain ID, with full details provided in Table S1. Data represent the mean of *n* = 3 replicates. Error bars are omitted for visual clarity to emphasize the proportional distribution.

To test the method’s universality, we then quantified stabilisomes from seven thermophilic cyanobacterial samples, which notably included both pure and mixed cultures (Table S1). While the absolute proportions of the major components varied significantly between strains, a consistent core chemical composition dominated by polyP, proteins, and amino acids was retained across all seven sources, regardless of whether the starting material was a monoculture or a mixed consortium (Figure 7). Achieving such high compositional consistency, especially from complex, multi-species samples, is a significant challenge in subcellular fractionation; for instance, purifying cyanobacterial phycobilisomes is often hampered by membrane contamination that alters the final product’s composition [32]. Therefore, the ability of our method to consistently yield a product with a conserved core composition from such diverse starting materials provides evidence for its universality.

Beyond universality, a truly robust method must also be sensitive enough to preserve genuine biological differences, which defines its high-resolution capacity. Our method also meets this standard. We observed significant variations in the component proportions between strains; for instance, the polyP content fluctuated from 21% to 36%. These variations are consistent with known biological variability [64,65,66]. This resolving power is critical in organelle research. Protocols for isolating magnetosomes, for example, often struggle to differentiate between developing and mature granules, yielding an averaged mixture that obscures true biological variability [67].

This quantitative compositional profile provides the first key experimental evidence for inferring the biological roles of stabilisomes. First, the high polyP content of nearly 30% suggests the stabilisomes’ central role as a primary cellular reservoir for both phosphate and high-energy chemical bonds, which is critical for maintaining nutrient and energy homeostasis in oligotrophic hot springs [12]. Second, the remarkably high abundance of amino acids (~14%) suggests a dual function. They likely serve as the primary counter-ions to balance the immense negative charge of the polyP backbone, a feature reported in other polyP-rich vacuoles [53], and also act as compatible solutes for protection against osmotic stress, a highly conserved strategy in microorganisms [60]. Finally, the substantial protein content (~20%) indicates that stabilisomes are not a passive storage particle but likely a densely packed hub of enzymatic reactions, a conclusion consistent with the diverse enzyme profile identified in our proteome.

However, it is important to note that the functional implications discussed above are inferred from structural and compositional data. While the present study establishes the chemical architecture, direct functional assays regarding thermal resilience were not performed in this work. Therefore, we propose the following hypotheses as a framework for future investigation: First, regarding thermal adaptation, we postulate that the high abundance of polyphosphate may function as a “chemical chaperone” to stabilize cellular proteins against heat-induced aggregation, while the specific lipid-protein interface preserves organelle integrity. Future studies employing in vitro thermal denaturation assays will be necessary to strictly verify this protective mechanism. Second, regarding biotechnological potential, the intrinsic stability and biological origin of these granules suggest promising applications as biomaterials. In contrast to synthetic polyphosphates, these biogenic nanogranules offer a natural interface that may yield superior biocompatibility. We envision future research exploring their utility as stabilizers for industrial enzymes or as bioactive scaffolds in tissue engineering. In summary, this study thus provides a critical link between fundamental organelle biology and the development of next-generation thermophilic bioresources.

## 4. Conclusions

In this study, we have successfully established and validated an innovative, structure-preserving, multi-step purification strategy that provides a novel solution for the high-purity isolation of stabilisomes from thermophilic cyanobacteria. In a direct comparison with conventional methods, our innovative protocol boosts purification efficiency by up to 10,000-fold, achieving a final granule-to-protein ratio of 1.061 × 10^7^ granules/μg and unequivocally demonstrating its superiority in achieving high purity. We further assessed the high integrity of the resulting bioproduct through multi-dimensional characterization. The isolated stabilisomes were highly homogeneous in morphology and size, with intact structures. The structural integrity and biochemical complexity of the purified stabilisomes revealed a conserved molecular architecture centered on polyphosphate scaffolds, supported by proteins, amino acids, and lipids. Furthermore, comparative analysis of seven different thermophilic cyanobacterial samples revealed that this core chemical composition is highly conserved among species, yet exhibits significant variability in the abundances of their major components, suggesting a dynamic adaptive adjustment. In summary, this work provides a fully validated methodological platform for the high-purity isolation of cyanobacterial stabilisomes. This technological breakthrough not only enables the first comprehensive chemical characterization of this organelle but also lays a critical foundation for future mechanistic studies on thermal resilience and the development of novel thermostable biomaterials.

## Figures and Tables

**Table 1:** The granules concentration and ratio of granule to protein by different separation methods.

Separation Methods	Number of Granules (Granules/mL)	Total Protein (μg/mL)	Granule-to-Protein Ratio (Granules/μg)	Fold Enrichment
Strategy A	(1.17 ± 0.2) × 10^7^	(1.176 ± 0.12) × 10^4^	(9.949 ± 0.12) × 10^2^	1 (Baseline)
Strategy B	(9.12 ± 0.12) × 10^7^	(2.56 ± 0.12) × 10^3^	(3.563 ± 0.12) × 10^4^	~358×
Strategy C	(8.35 ± 0.15) × 10^9^	(7.872 ± 0.12) × 10^2^	(1.061 ± 0.12) × 10^7^	~10,664×

This table compares the purification efficiency of the three different separation methods. The comparison is based on the final concentration of isolated granules, the amount of co-purified total protein, and the calculated granule-to-protein ratio, which serves as a metric for purity. Data are presented as mean ± SD (*n* = 3 independent biological replicates).

**Table 2:** Key protein components identified in stabilisomes from the representative strain, *Synechococcus* sp.

Protein Description	Clusters of Orthologous Groups (COG)_Function_Description	Kyoto Encyclopedia of Genes and Genomes (KEGG)_Description
ATP synthase subunit a	ATP binding	Oxidative phosphorylation
Phosphate-binding protein	ABC-type phosphate transport system, periplasmic component	Phosphate transport system substrate-binding protein
PPKa	polyP biosynthetic process	Inorganic ion transport and metabolism
Histidine kinase	L-serine biosynthetic process	L-phosphoserine phosphatase activity
Agmatine deiminase family protein	Putrescine biosynthetic process	Amino acid transport and metabolism

Functional annotation of representative key proteins identified in the stabilisome proteome. Protein functions were categorized based on the COG database and mapped to metabolic pathways using the KEGG database [42,43,44]. a PPK, polyphosphate Kinase.

## Data Availability

The metabolomics data generated and analyzed during the current study are available in the MetaboLights repository with the dataset identifier MTBLS13010 (https://www.ebi.ac.uk/metabolights/MTBLS13010). The mass spectrometry proteomics data have been deposited to the ProteomeXchange Consortium via the PRIDE partner repository with the dataset identifier PXD068468 (https://www.ebi.ac.uk/pride/archive/projects/PXD068468 (accessed on 1 February 2026)).

## Supplementary Materials

The following supporting information can be downloaded at: https://www.mdpi.com/article/10.3390/plants15030499/s1, Figure S1: Light microscopy images of the seven thermophilic cyanobacterial strains used in this study; Figure S2. Representative MS/MS spectral mirror plots for key metabolites; Table S1: Thermophilic cyanobacterial strains used in this study and their native habitat information; Table S2: Complete list of proteins identified in the stabilisome proteome; Table S3: Complete list of metabolites identified in the stabilisome metabolome.

## Abbreviations

The following abbreviations are used in this manuscript:

EDS	Energy-dispersive X-ray spectroscopy
NTA	Nanoparticle tracking analysis
GO	Gene Ontology
KEGG	Kyoto Encyclopedia of Genes and Genomes
COG	Clusters of Orthologous Groups
LC-MS	Liquid chromatography–tandem mass spectrometry
ICP-MS	Inductively coupled plasma mass spectrometry
ELISA	Enzyme-linked immunosorbent assay
PCA	Principal component analysis
polyP	Polyphosphate
PG	Phosphatidylglycerol
PE	Phosphatidylethanolamine
TEM	Transmission electron microscopy
MWCO	Molecular weight cut-off
PPK	Polyphosphate kinase
TCA	Tricarboxylic acid cycle
ADP	Adenosine diphosphate
AMP	Adenosine monophosphate
ATP	Adenosine triphosphate
BCA	Bicinchoninic acid
DDA	Data-dependent acquisition
DIA	Data-independent acquisition
DTT	Dithiothreitol
SD	Standard deviation
ANOVA	Analysis of variance
UHPLC	Ultra-high-performance liquid chromatography
