# Supplementary material for: High-Purity Isolation of Polyphosphate-Rich Stabilisomes Defines Their Conserved Chemical Architecture in Thermophilic Cyanobacteria
Source: Plants (Basel). 2026 Feb 5;15(3):499. doi: 10.3390/plants15030499 (PMC12899096; doi:10.3390/plants15030499)
Supplement: Supplementary file 1 [file plants-15-00499-s001.zip › Supplementary_Information.pdf]

Supplementary Table S1. Thermophilic cyanobacterial strains used in this study and their native habitat information.

Strain ID	Strain Name	Sampling Location	Temperature (°C)	pH
FJSJ-1	*Synechococcus* sp.	Shajian Hot Spring, Fujian	52°C	6.7
GDSG-1	*Synechococcus* sp.	Shaoguan Hot Spring, Guangdong	67°C	6.5
GDSG-2	*Synechococcus* sp.	Shaoguan Hot Spring, Guangdong	83°C	6.5
JLCBS-1	*Nostoc* sp.	Changbai Mt. Hot Spring, Jilin	74°C	7.0
GDMZ-1	*Lyngbya* sp.	Meizhou Hot Spring, Guangdong	50°C	6.7
GDSG-3	Mixed Culture (*Anabaena* sp. and *Synechococcus* sp.)	Shaoguan Hot Spring, Guangdong	46°C	6.2
GDSG-4	Mixed Culture (*Anabaena* sp. and *Synechococcus* sp.)	Shaoguan Hot Spring, Guangdong	65°C	6.5

This table provides the strain identifier (Strain ID), species name (Strain Name), geographic sampling location, and the in-situ measured temperature and pH for each cyanobacterial strain.

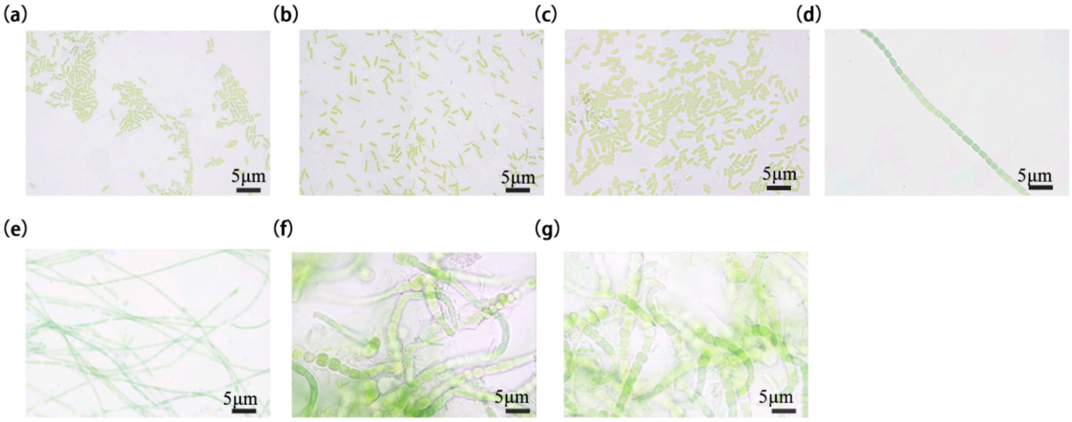

Supplementary Figure S1. Light microscopy images of the seven thermophilic cyanobacterial strains used in this study. Representative light micrographs showing the typical morphology of the seven thermophilic cyanobacterial samples listed in Table 1. The corresponding Strain ID for each panel is: (a) FJSJ-1 (*Synechococcus* sp.), (b) GDSG-1 (*Synechococcus* sp.), (c) GDSG-2 (*Synechococcus* sp.), (d) JLCBS-1 (*Nostoc* sp.), (e) GDMZ-1 (*Lyngbya* sp.), (f) GDSG-3 (*Anabaena* sp. and *Synechococcus* sp.), and (g) GDSG-4 (*Anabaena* sp. and *Synechococcus* sp.). All images were taken at the same magnification. Scale bar = 5 μm.

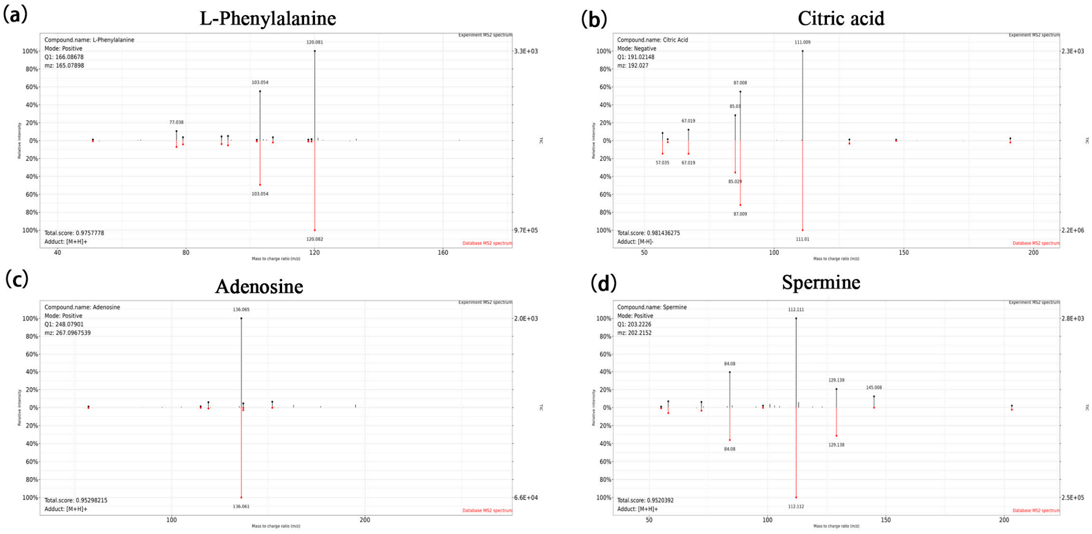

Supplementary Figure S2. Representative MS/MS spectral mirror plots for key metabolites. The upper panels (black) display the experimental MS/MS spectra acquired from stabilisome samples, while the lower panels (red) represent the reference spectra from the Metware Database (MWDB). The high degree of spectral similarity and matching scores confirm the structural annotation (MSI Level 2) for these representative compounds. (a) L-Phenylalanine; (b) Citric acid; (c) Adenosine; (d) Spermine.
